# Combination Fillings

**Published:** 1892-03

**Authors:** J. R. Callahan

**Affiliations:** Cincinnati, O.


THE DENTAL REGISTER.

Vol. XLVI.] MARCH, 1892. [No. 3.

### COMMUNICATIONS.

### Combination Fillings.

BY J. R. CALLAHAN, D.D.S., CINCINNATI, O.

Read before the Ohio State Dental Society, held at Columbus, O., December, 1891.

For sometime I have been deeply impressed by the seeming tendency, shown by many of the leading lights of our profession, to the utter abandonment of things practical for things scientific, so-called. For a time the energies and best thoughts of these men were directed toward the practical things in hand, and *at the same time* they did not fail to recognize the scientific; and while working under these conditions they pushed American dentistry to the front and compelled other nations to acknowledge their superiority. As an indication of the tendency of the times, I read in one of our journals not long ago an article congratulating the profession at large upon the fact, that at a recent dental society meeting, that not a practical paper or discussion was heard; that the whole time had been devoted exclusively to scientific subjects. To all of which I say, Amen, if kept within proper bounds. There is in my mind no doubt as to the propriety of devoting the whole of the time, of such societies as the American Dental Association, to scientific discussions and demonstrations. But our State societies should, in my opinion, divide the time somewhat and discuss and *demonstrate* the things of every day practice. Our hyper-scientific men, especially those who ape European practice, seem to have forgotten that there is any necessity for doing practical things thoroughly and up to the highest standard. They seem to forget that their *principal* mission is to save teeth, at least a look into the mouths of their patients would lead a conscientious operator to think so; for this reason, be it real or imaginary, when your committee called on me for a paper I concluded to direet your attention, *in a very* general way, to combination fillings.

Human opinions and methods seem to swing as a pendulum, from one extreme to the other, until, after a lapse of time we are compelled by results to seek the middle ground where we may look upon all sides of the problem in hand, thereby be enabled to recognize that which is good and discard that which is bad. Many of you can remember when fillings were all plastic or non-cohesive. All of us can remember when cohesive fillings were the rage. To-day the man who confines his practice to either extreme may be said to be a one-sided dentist. We seem to have found the middle ground in this department, and are doing our patients and our profession better service than ever before.

For our purpose to-day combination fillings will mean a combination of amalgam and gold, tin and gold, and of cohesive gold and non-cohesive gold.

It is well known to all present that the rapidly increasing weakness of the enamel borders as we approach the cervical line, calls, and calls loudly for an indestructible filling material that will spread under gentle pressure and make a perfect joint and that will not change its form during years of service. Another requirement also, is that the material must be of such nature that it can be put in place with the least possible expenditure of time consistent with perfect results.

For the sake of brevity we desire to direct your attention entirely to the filling of compound cavities as we find them in bicuspid and molar teeth, with the understanding that the grinding surfaces of these fillings are to be made of cohesive gold only. I do not need to describe the preparation of the cavities to you further than to say, the borders should be cut away till we have as strong and even walls as possible, with a very slight groove cut along the lingual and buccal walls and the base of the cavity, or in other words, the cervical wall should be made square as the case will permit, without making sharp angles, thereby giving the filling a firm foundation to rest upon. We should get the chief anchorage at or near the grinding surface, in most cases in the fissures.

It is absolutely necessary with this kind of filling to use some form of matrix, whatever kind best suits the oprator. In my opinion the matrix should be so adjusted that it will give a little, so that the filling may overlap the borders slightly. Now we proceed to fill as our judgment may direct, first with amalgam filling one or two-thirds of the approximal portion of the cavity with whatever may be your favorite brand of amalgam, then laying on the crystalloid gold prepared for this purpose, till all the mercury disappears and we have the clean gold surface. When the remainder of the cavity is filled with cohesive gold, this makes a very good filling. Under these conditions amalgam is seen to the best advantage. I have seen fillings of this combination doing excellent service for years, the amalgam shrinking much less, therefore, being far more reliable than when used alone.

If it is desired to use pure tin in the approximal portion of the cavity, we have the choice of two forms of tin, viz: tin foil and Robinson's Fibrous Filling Material. The latter being so far the superior of the former we take the liberty of passing the foil by without further notice.

With the Robinson material the mallet must be used and used very thoroughly, with it you can go to almost any extreme in contouring. It will spread somewhat under the plugger, soon turns dark but does not discolor the tooth substance, and if thoroughly condensed and properly finished, will keep a tooth free from decay, under unfavorable circumstances, longer than any single metal used for that purpose, provided it be protected from friction such as the grinding surfaces are exposed to. By way of experiment to test the strength of this material I have here to show you a very large crown restoration, built up of Robinson's material and then a layer of gold foil tacked over the exterior surfaces giving it the appearance of a solid gold filling. I have similar fillings in the mouths of a few patients simply to see how they will stand the wear and tear. They have been in use now nearly ten years and when last examined, about a year ago, they were practically as perfect as the day they were put in. The filling to which I have given the preference for several years is composed of equal parts of tin and gold for approximal surfaces and finished with heavy cohesive gold. The material is prepared by laying a sheet of Abbey's No. 4 non-cohesive gold foil on a sheet of No. 4 tin, and folding once, with the tin on the outside; then cut in strips from $\frac{1}{8}$ to $\frac{1}{4}$ inch in width; then introducing the material by hand pressure, using rather coarse and sharp serrations, once in a while bringing the mallets into use to make sure the filling is solid as possible. I have been told by dental friends that this sort of filling would disintegrate and finally wash out from under the gold, and I must confess that for a time I was quite uneasy about the large number of these fillings I had put in, but after several years of close observation I find if there is any disintegration it has been where the filling had not been made solid when put in. After the tin and gold has been in the tooth a short time a change begins to take place in the filling material. What it is I do not pretend to say, only that the material gets quite hard, as hard as amalgam. It can be separated from the gold only by cutting. There is no change of form whatever that I have been able to discover. As to strength it is perfectly safe to restore any ordinary contour. I have here an extreme case to show you that was built entirely by hand pressure, using the mallot only on the grinding surface. For a long time I have used this material for filling cavities on grinding surfaces of molars and bicuspids for children, they do so well and show so little wear that I am tempted to use them for adults also.

I have tried many times to use non-cohesive gold in these approximal surfaces in this manner without achieving what I felt to be a success, the gold always showing a disposition to harden and get lumpy under pressure and scale in finishing. Recently my attention was called to the Wolrab pellets; have been giving them a pretty thorough trial for about a year and so far as I can see now they fill the bill admirably. It spreads under the instrument, either hand or mallet, and does not show that harsh and brittle form so characteristic of other gold. I do not think it will make as safe a contour as Robinson's material, nor will it work quite so fast as other materials mentioned; but where cavities are exposed to view and the preparations of tin are barred out on account of color, you will find these pellets will make a quick and very reliable filling under a few layers of No. 60 cohesive foil. I have avoided some details that I consider to be of some importance for the reason that I expect to put in one or more of these fillings in your presence during this meeting.

---

DISCUSSION ON DR. CALLAHAN'S PAPER.

*Dr. C. M. Wright:*—I think the paper read by Dr. Callahan is a good one of its kind. I will not say any thing about the kind. He started in with a criticism of a society of dentists who had nothing but scientific papers presented. Once upon a time there was a lot of griddle-makers in the country who devoted themselves to the mechanical operation of making griddles for the people. The principle thing was to make good griddles so that the people might make cakes in the morning. But the griddle-makers got to studying the qualities of the material used in the cakes; they studied also the subject of nutrition and general biology, and little by little, instead of discussing the best methods of making griddles when they met in griddle-makers' convention, they talked about the health of the people who ate the griddle cakes, until after a while they established a school, and were recognized as a profession. By and by a wicked man by the name of Callahan came along, not caring about the health of the people, nor the kind of cakes eaten, nor the questions of physiology, and said, "Let us confine ourselves to the manufacture of fine griddles, this is practical." This is Dr. Calihan's position in our society to-day.

*Dr. Callahan:*—The remarks of Dr. Wright are to the point, as they usually are. So far as devotion of time to scientific discussion and work is concerned, I do not think there is any man who spends more time from this standpoint than I do. In my opinion the scientific work should be better, and the class of practice I have reference to many of you are familiar with. Many of you see the work of men who occupy prominent positions in our profession who can do better work than they do, but they send their patients away as if they thought it was not necessary to put in a good filling. We should pay a little more attention to this line. When I read the paper I had in mind two or three men in New York City, one the president of a dental college there, whose work his patients are not satisfied with. Much better work could be done than is done if proper care were taken to do it.

*Dr. H. A. Smith:*—This is too important a subject to let go without further discussion. Dr. Callahan has gone into the subject forcibly and presented his reasons why combination fillings should be often made. He did not refer perhaps to the economic side of the subject. There are several reasons why these fillings might be made from that standpoint. Should we estimate the cost of material? Some one asked not long ago the relative cost between the cheaper materials and gold? Taking the silver dollar as a basis, it was stated "the relative cost would be about as one to thirty." From that standpoint perhaps we ought not to insert them. If there is to be any advantage, however, in this direction it should be largely on the side of the dentist. If a filling of one form of gold, say soft gold, could be introduced into a cavity and regarded as a thorough filling, it is just as good as a combination filling and, therefore, I think, a combination of materials is sometimes resorted to, as Dr. Callahan has intimated, because the operator does not exercise or possess the requisite skill to make good gold fillings. Permit me to call attention to a combination of the metals and oxy-phosphate. I have in my hand a combination filling composed of one-half in bulk of what is called Leslie's crystaline gold and oxy-phosphate. These fillings I have inserted occasionally, but can not give any thing definite in reference to their durability. The filling presents a dense surface; it has the advantage of edge and strength, and for these reasons it might be preferred. Whether it is dissolved as rapidly as a phosphate filling I do not know. It seems to me we would not have a solution of the surface as readily. The gold can be burnished over the surface and this would be a protection to prevent the beginning of the solvent action. This may not be new to you. We have had our attention frequently called to combination fillings in which the metals are associated with oxy-phosphate. I have here specimens, fillings made of oxy-phosphate and alloy, such as are used in amalgam fillings. They are objectionable in that they discolor. They have two qualities, however, in their favor, density and edge-strength qualities, which we do not have in oxy-phosphate fillings. I tried the experiment the other day of introducing an oxy-phosphate filling and placing immediately upon the surface a mat of spoige gold; it seemed to adhere. After hardening the gold was condensed upon the surface and we had a phosphate filling with a gold surface. If the cavity is accessible and the manipulation done rapidly this might be accomplished.

*Dr. C. P. Dennis*, Portsmouth. This subject is too important to permit it to go by without some discussion as it is one that we have to deal with in our daily practice. I will undertake to say that it is much like our religious duties, there is so much difference between the profession and our conduct in the matter. It is a very important matter also, because the durability of our operations in the mouth depend so largely upon the condition of the mouth and teeth, and the care that is taken of them. I try to impress upon every patient who comes into my office the necessity of caring for the teeth, and when I have not got time to talk about it I give them printed directions. It seems to me we can not expect operations to be lasting unless the mouth and teeth are kept in a healthy condition. In the press of business of attending to our patients we forget these thing, and as I have before said, I keep slips containing directions near by me so I can hand them to patients as I discharge them. Dr. Taft has covered the ground so thoroughly that there is very little left to be said. I commend the paper because it tells us to do our duty rather than what our duty is.

*Dr. W. H. Whitslar*, Cleveland. There was a discussion at a previous session of the society on the care of the teeth during pregnancy and lactation and it strikes me that this subject would have come under that head very nicely. The means of proper food, the proper filling of teeth and care of the mental state were touched upon in that discussion but nothing, to my knowledge, was said in regard to the teeth from a hygienic standpoint. It has been my experience that women during the period of pregnancy and lactation have so much to do in taking care of children that their minds are constantly occupied in that direction, consequently they neglect their teeth. If they had taken care of them, provided they were of good structure, they would not have decayed. We must not at all times say that teeth decay because they lose their lime salts, or because they are not properly filled. Care of the teeth during pregnancy and lactation by the proper means, the use of the brush, etc., will prevent them from decaying in a great many instances.

*Dr. J. Taft*:—It strikes me that this is a matter of importance both to the dentist himself and the profession, because the greater efficiency he can secure in that which he does for his patients in the way of arrest of decay of the teeth the better will he be held in the estimation of those whom he serves. It is his duty from the standpoint of self-preservation so far as professional reputation is concerned and it is his duty to give such directions as will secure the most perfect results in this respect. There are various conditions in life; there is a lack of care on the part of people in regard to their teeth. Sometimes there may have been much work done in the mouth of the patient, and he has received no instruction about their care, and by and by he or she becomes negligent and an accumulation of debris is made upon the teeth and remains for an indefinite period of time; indeed all of you have seen cases of the first, second and third molar where there has been an accumulation of material upon the surface undergoing decomposition, and you can almost predict in many instances that beneath this accumulation there is a disintegration of tooth substance. If a filling has been put into that surface of the tooth you will almost instinctively come to the conclusion that the condition is faulty, and I suspect about that filling there is decay. In a great many instances it will be found that disintegration takes place there. Now then, it is important for the dentist from a professional standpoint, that he should pursue that course in the way of impressing upon the minds of his patients the necessity for care in this respect for the preservation of their teeth.

Another point. Every one who is a regular patient should have his or her mouth examined at certain intervals every six months on an average. Some patients ought to have their teeth examined every three months. The dentist should see that the patient is careful in this matter, if possible, and not allow him or her to lapse into careless habits; he should see that they are masticating their food well and that there are no extensive breaches made in the teeth. Other patients will go along six months without an examination of their teeth being necessary, and still others nine months, and you feel satisfied to let them go that long, knowing that they are careful in their habits. Another patient of different habits will have large decay made within a year's time. The character of the teeth, their susceptibility to disease, modify the circumstances relative to the frequency with which the teeth should be examined. The dentist who examines the teeth of regular patients should see that every filling is in good condition all the while. Fillings should be gone over and kept in good condition, for there is no reason in the world why fillings may not be impaired by use in three or four years, by abrasion or roughness produced in various ways. When we get a hole in our boot we have it repaired; when we get a hole in our coat we have it mended, but teeth oftentimes go along without being touched. Teeth should be examined in this respect, and we should insist upon those who hold us responsible for their teeth that these examinations shall be made. A patient holds you responsible for what you have done.

We hear it said sometimes "Dr. So-and-So filled my teeth and they are all going to pieces." Insist upon seeing the cases frequently and do whatever is necessary to maintain a perfect condition of the mouth. That is due to the dentist. As he builds up his reputation, so he is established in the community; it is a duty we owe both to ourselves and patients.

